# A Robust Machine Learning Based Framework for the Automated Detection of ADHD Using Pupillometric Biomarkers and Time Series Analysis

**DOI:** 10.1038/s41598-021-95673-5

**Published:** 2021-08-12

**Authors:** William Das, Shubh Khanna

**Affiliations:** 1 Columbia University, New York, NY 10027 USA; 2 Stanford University, Stanford, CA 94305 USA

**Keywords:** Predictive medicine, Data mining, Statistical methods, Diagnostic markers, Machine learning

## Abstract

Accurate and efficient detection of attention-deficit/hyperactivity disorder (ADHD) is critical to ensure proper treatment for affected individuals. Current clinical examinations, however, are inefficient and prone to misdiagnosis, as they rely on qualitative observations of perceived behavior. We propose a robust machine learning based framework that analyzes pupil-size dynamics as an objective biomarker for the automated detection of ADHD. Our framework integrates a comprehensive pupillometric feature engineering and visualization pipeline with state-of-the-art binary classification algorithms and univariate feature selection. The support vector machine classifier achieved an average 85.6% area under the receiver operating characteristic (AUROC), 77.3% sensitivity, and 75.3% specificity using ten-fold nested cross-validation (CV) on a declassified dataset of 50 patients. 218 of the 783 engineered features, including fourier transform metrics, absolute energy, consecutive quantile changes, approximate entropy, aggregated linear trends, as well as pupil-size dilation velocity, were found to be statistically significant differentiators (*p* < 0.05), and provide novel behavioral insights into associations between pupil-size dynamics and the presence of ADHD. Despite a limited sample size, the strong AUROC values highlight the robustness of the binary classifiers in detecting ADHD—as such, with additional data, sensitivity and specificity metrics can be substantially augmented. This study is the first to apply machine learning based methods for the detection of ADHD using solely pupillometrics, and highlights its strength as a potential discriminative biomarker, paving the path for the development of novel diagnostic applications to aid in the detection of ADHD using oculometric paradigms and machine learning.

## Introduction

Attention-deficit/hyperactivity disorder (ADHD) is a clinically heterogeneous neurobehavioral disorder characterized by inattention, impulsivity, and hyperactivity^1^. Its overall prevalence among children and adolescents is 5–8%, affecting upwards of 1 million children per year in the US alone^2^. The long-term effects of untreated ADHD are detrimental for individuals and their families. Children and adults with untreated ADHD suffer from poor educational outcomes and familial relationships^3^, as well as increased economic burdens. Moreover, untreated ADHD during childhood is a risk factor for later adult mental health issues^4^. Lack of treatment impairs social and occupational functioning and increases the likelihood of developing comorbid disorders such as heightened anxiety, depression, personality disorders, and antisocial behaviors^5^. Additionally, inaccurate clinical evaluations can lead to inappropriate treatment interventions, such as incorrectly administering stimulant drug medications that have side effects on healthy children^6,7^. Accurate and efficient diagnosis of ADHD, thus, is crucial to effectively administer treatments and prevent subsequent complications for a child’s socioemotional development, academic and occupational achievement, and overall welfare.

### Current Diagnosis

Current clinical diagnosis is subjective, inefficient, and inaccurate. There is no reliable objective test to diagnose ADHD^8,9^, as diagnosis is based solely on observed behavior and reported symptoms, creating a risk of over and under-diagnosis^9,10^. Evaluations are based on a checklist of eighteen symptoms, nine related to inattention and nine related to hyperactivity and impulsivity^11^. These subjective clinical assessments often last multiple hours^6^. Moreover, the demand for these examinations greatly exceeds the maximum capacity of available developmental pediatric clinics^6^. As a result, children are often waitlisted for over a year, preventing timely diagnosis and delaying the start of necessary treatment^6^. Wait times can extend beyond 13 months for minorities or socioeconomically disadvantaged groups^6^. Other assessment methods such as computerized performance tests have been criticized due to poor clinical utility^12^. Medical experts collectively agree that the lack of an objective and efficient mechanism to characterize ADHD remains a pervasive and detrimental problem, precluding effective treatment regimens^3^.

### Pupillometric Variation in ADHD

Due to the currently deficient methods for diagnosing ADHD, a reliable objective mechanism to characterize the disorder is necessary to ensure accurate and efficient diagnosis. Oculomotor paradigms are particularly adept in analyzing abnormalities in brains affected by neurodevelopmental disorders^13–16^. Pupillometric-based analyses have been applied to a number of psychiatric disorders, such as obsessive compulsive disorder, autism, dyslexia, and Tourette syndrome, in order to gain behavioral insights into correlations between eye movements and behavior^13,17,18^. In particular, one promising biomarker for ADHD in humans is pupil-size dynamics—the ways in which the pupil responds to certain visual stimuli^9^. Pupil-size dynamics have been shown to be associated with the brain norepinephrine system, an area which controls executive functioning^19^ and is impaired by ADHD. In accordance with this, Wainstein et al. showed in an experimental study that pupil-size dynamics were strong differentiators between ADHD positive and negative subjects after performing statistical analysis; moreover pupil-size was also shown to be strongly correlated with attentional performance in subjects^9^. Geng et al. showed that pupil size reflects uncertainty in users who completed a visuospatial working memory task^20^. Wahn et al. additionally showed that pupil-size dynamics can be utilized as a reliable metric to assess attentional load in patients^21^. Given the vast literature highlighting associations between pupil-size dynamics and attentional performance in neurobehavioral disorders, we hypothesized that pupillometric features could be utilized as objective biomarkers to effectively characterize ADHD using a machine learning based and time series analysis methodology.

### Applications of Machine Learning

Babiker et al. developed a machine learning model to predict a user’s emotional state using pupillometrics, engineering a set of features based on pupil-size dilation velocity and acceleration; their method achieved overall 96.5% accuracy, 97.93% sensitivity, and 98% specificity^22^. Similarly, Qian et al. devised a machine learning-based model to classify visual responses based on pupillometrics^23^. Baltaci et al. also incorporated a machine learning-based model to to classify user’s stress response and mental state based on pupillometrics, extracting a variety of statistical features based on pupillary movement^24^.

As such, we sought to develop a machine learning-based framework to analyze pupillometric variation in subjects, hypothesizing that it would accurately discriminate between ADHD positive patients and control subjects. Using the engineered features, we also sought to extract valuable oculometric patterns to advance current understandings of pupillometry regarding the presence of ADHD using various feature visualization techniques and statistical validation.

## Dataset

The preprocessed time series dataset evaluated in this study was released by Wainstein et al., conducted under Universidad Católica de Chile^25,26^. The subjects were elementary school children aged 10–12, recruited from local schools in Chile^25^. Three specific groups of subjects are included: *Off-ADHD, On-ADHD* and *Ctrl*^9^. *Off-ADHD* corresponds to positively diagnosed subjects not taking any medication, while *On-ADHD* corresponds to those taking medication. *Ctrl* represents a control group of healthy subjects. The 28 *Off-ADHD* subjects consisted of 4 girls, 24 boys, and the 22 *Ctrl* subjects consisted of 4 girls, 18 boys^25^. The medication administered was methylphenidate. The Eyelink 1000 device was used to record oculometrics at a sampling frequency of 1 kHz^25^. More specific experiment details are outlined extensively in Wainstein et al.’s publication^25^.

We assess model performance for binary classification by considering the ADHD Positive Group with only *Off-ADHD* ground truth labels included. We note that the inclusion of on- and off-medication subjects introduces a bias in the ADHD Positive class through a confounding variable of receiving medication; this bias would not accurately reflect the population of patients with ADHD, as medication regulates the symptoms of ADHD and potentially impacts pupillometric patterns. This would degrade the validity of the machine learning algorithms if they were to be trained and tested on biased data. As such, the ADHD Positive class comprised of only *Off-ADHD* subjects, amounting to a full dataset of 50 instances.

### Experiment Details

A group of 50 subjects participated in the study. 28 subjects were patients diagnosed with ADHD, and 22 were healthy control patients. A subgroup of 17 ADHD patients performed the task twice, on and off medication, denoted as *On-ADHD* and *Off-ADHD* subjects, respectively^25^.

All subjects were required to complete a visuospatial working memory task, which consisted of multiple 8 s trials, during which pupil-sizes were measured. In the first five seconds, three dot arrays were presented, followed by a distractor image. In the last three seconds, subjects were presented with a random dot array and asked to determine if the dot had been presented to them before^25^.

## Methods

### Feature Engineering

Due to the high dimensional nature of the time-series data—each patient consisted of 160 trials, each containing 8000 timestamps of data—in order to reduce computational costs and ensure time-efficient analysis for feature extraction, for each trial, the time-series data was reduced to 500 timestamps by taking a moving average window of size 16ms and concatenating the results. We found that the overall structure of the time-series data was retained from the average sliding window approach. A side-by-side figure of original and reduced time series is provided in the Supplementary Information.

A total of 783 features were engineered using time series analysis software—we engineered 22 custom features using garnered intuition on the pupillometric data. Engineered features obtained from software include aggregated linear trends, fourier transform metrics, energy spectral density, approximate entropy, as well as standard statistical values such as the mean, median, standard deviation, and variance of pupil-sizes during various intervals across the time-series. Features were calculated per-trial and then averaged across all trials, grouped by patient ID, in order to provide a thorough and encompassing feature space for each patient. Missing values were estimated using cubic spline interpolation prior to analysis, and trials with more than 80% of the data missing were excluded from analysis.

**Figure 1:**
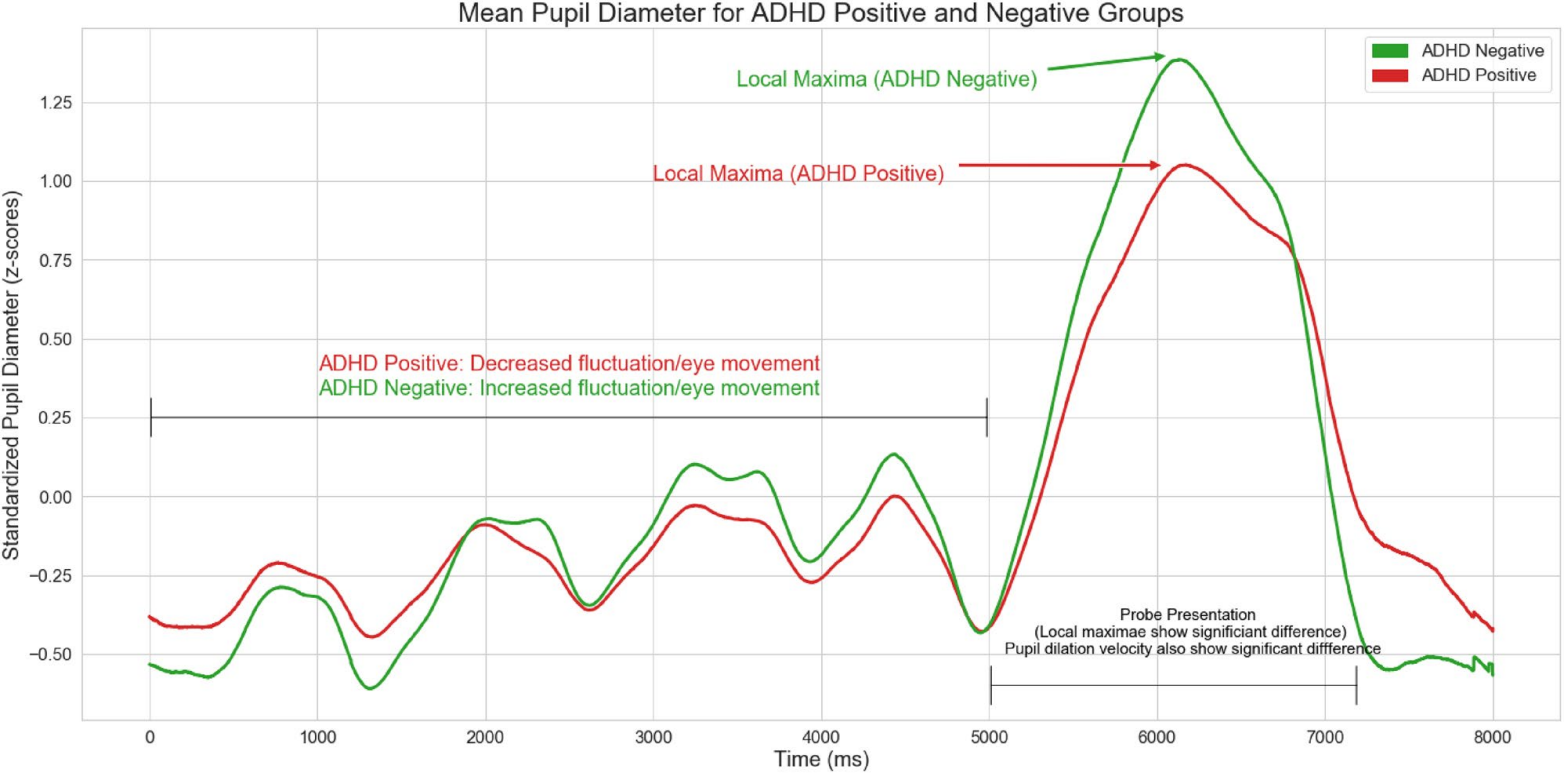
Time-series visualization of the mean pupil diameter averaged across multiple trials, grouped by ADHD diagnosis.

**Figure 2:**
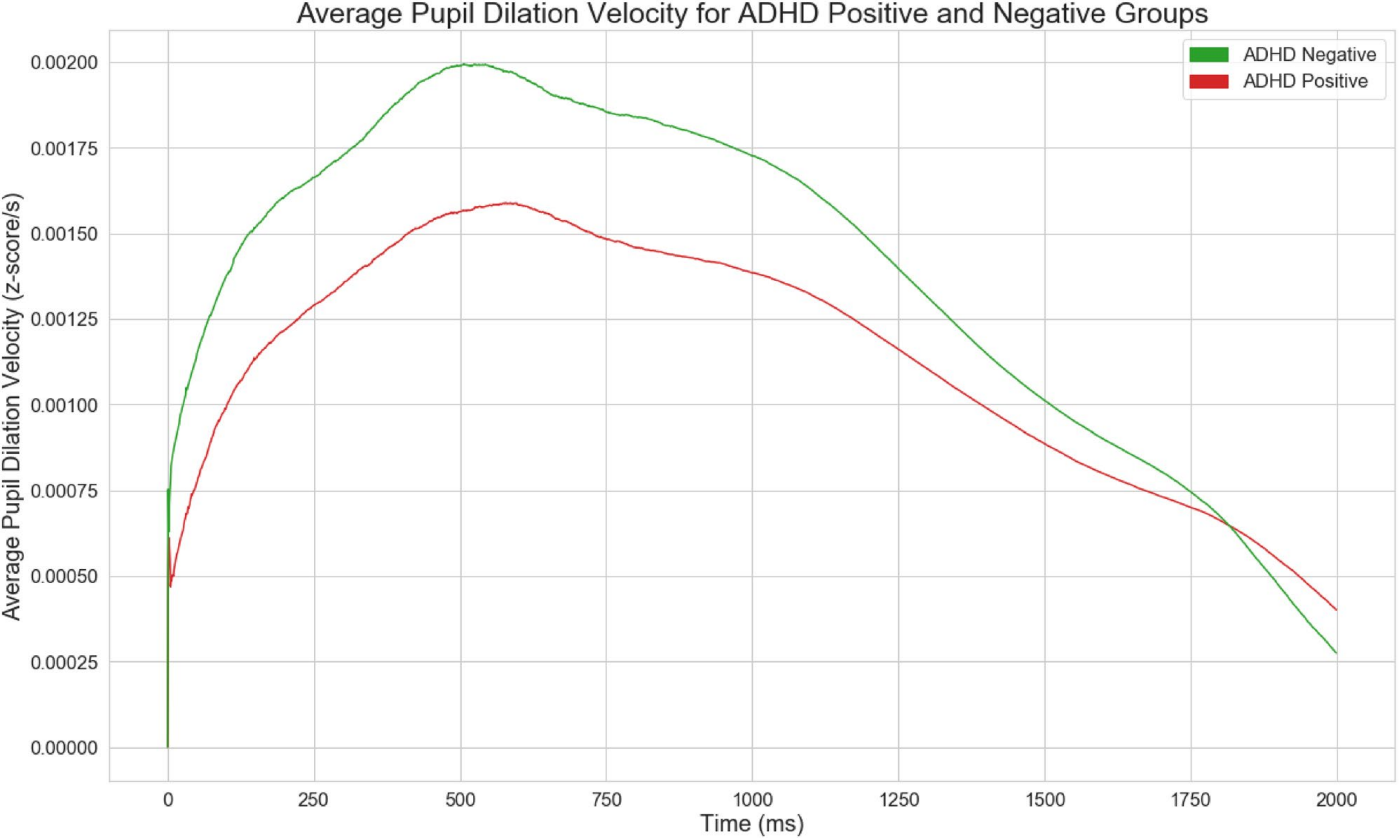
Pupil size dilation velocity following the presentation of a stimulus.

**Figure 3:**
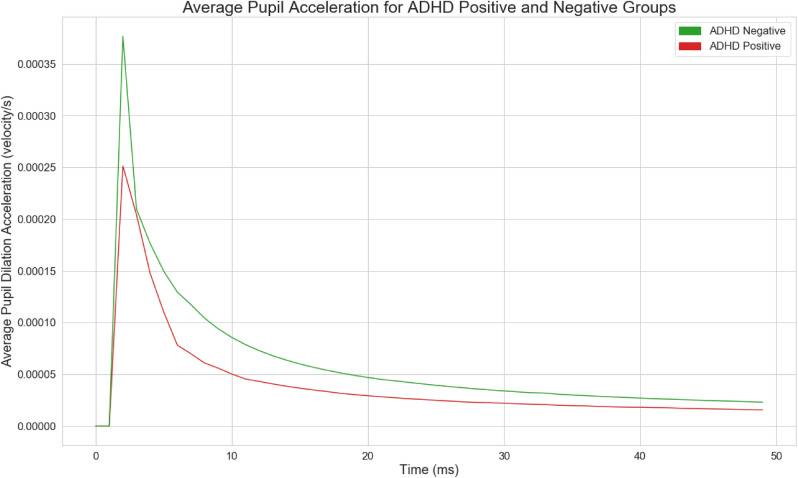
Pupil size dilation acceleration following the presentation of a stimulus.

For custom feature extraction, further building off of Wainstein et al.’s original visualizations, pupil-sizes for each group were averaged and visualized in Fig. 1 in order to extract novel and informative features. Patterns were visually observed and validated using statistical significance tests. Pupil-size dilation velocity and acceleration, two of the engineered features, were visualized per group as shown in Figs. 2 and 3, calculated from Fig. 1.

#### Pupil Size Dilation Metrics

We engineered custom features based on pupil-size dilation velocity and acceleration using garnered intuition on pupil-size dynamics between ADHD Positive and Negative groups. Let $P_S$ denote the the presentation of a probe at the 5000 ms mark, and let $P_{T_1 - T_2}$ denote the time interval from time $T_1$ to $T_2$ in ms, as shown in Table 1. Given a starting timestamp $T_0$ and pupil size $P_0$, the pupil size dilation velocity $V_i$ at any given timestamp $T_i$ with pupil size $P_i$ is given by:

1 $$\begin{aligned} V_i = \frac{P_i - P_0}{T_i - T_0}. \end{aligned}$$

The acceleration $A_i$ is given by:

2 $$\begin{aligned} A_i = \frac{V_i - V_0}{T_i - T_0}. \end{aligned}$$

The accumulated pupil-size dilation velocity $AV_i$ is the sum of: $V_0 + V_1 + \cdots + V_i$.

**Table 1:** Custom pupillometric feature extraction per trial.

1	Local maxima during $P_{5000-7000}$
2	Local maxima during $P_{1-5000}$
3	Local minima during $P_{6500-8000}$
4	Mean pupil size during $P_{5500-7000}$
5	Pupil size standard deviation
6	Pupil size kurtosis
7	Pupil size skew
8	Median pupil size
9	Mean pupil size
10	Mean pupil size dilation velocity before $P_S$
11	Mean pupil size dilation velocity after $P_S$
12	Max pupil size dilation velocity (MaxPSDV) before $P_S$
13	MaxPSDV after $P_S$
14	Max accumulated pupil size dilation velocity (MaxAPSDV) before $P_S$
15	MaxAPSDV after $P_S$
16	MaxAPSDV after $P_S$ − MaxAPSDV before $P_S$
17	MaxPSDV after $P_S$ − MaxAPSDV before $P_S$
18	Mean pupil size dilation $P_S$ − MaxAPSDV before $P_S$
19	MaxPSDV after $P_S$ − MaxASPDV before $P_S$
20	Max pupil size acceleration during $P_{5000-7000}$
21	Min pupil size acceleration during $P_{5000-7000}$
22	Mean pupil size acceleration during $P_{5000-7000}$

### Machine Learning Framework

#### Classification Algorithms

The following binary classification algorithms were trained using the engineered features: Logistic Regression, KNN Neighbors, Naive Bayes, Decision Tree Classifier, and a Random Forest Classifier. Since over-complex algorithms tend to overfit on small datasets, evaluation was limited to the aforementioned classifiers.

#### Model Evaluation

A comprehensive evaluation of each algorithm was conducted: due to the small dataset size, training and evaluating using a holdout set would not accurately reflect the generalization ability of a model and unnecessarily degrade predictive power by reducing training set size. As such, nested ten-fold cross-validation (CV) was employed to assess the performance of the models. Nested CV provides a robust and approximately unbiased estimate of test error: it addresses problems in traditional K-fold CV and leave-one-out CV, which have been shown to yield overly optimistic and biased test error estimates^27,28^. Nested CV is a reliable alternative to K-fold CV when estimates of the underlying hyperparameter search is desired—for the nested CV pipeline, an inner CV loop is used to tune hyperparameters for various models; the optimized model is then applied to a separate outer CV loop that assesses model performance without producing overly optimistic estimates. For feature selection per-fold, 9 optimal features were filtered using a standard univariate correlation-based one-way ANOVA F-test for feature ranking, which analyzes numerical input in conjunction with class labels. Since an excess of features can cause overfitting, especially for small datasets, a fixed value of 9 was arbitrarily established for selecting features in each fold for nested CV. Features were selected using only the train data in each fold in order to prevent overfitting and leaking of information into the test set. The nested CV procedure was repeated for a total of 30–50 times for each model, enabling the construction of confidence intervals for binary classification metrics.

Standard medical diagnostic metrics—sensitivity (true positive rate), specificity (true negative rate), area under the receiver operating characteristic (AUROC), as well as accuracy— were taken into account for analysis of model performance based on nested CV.

#### Feature Intuition

**Figure 4:**
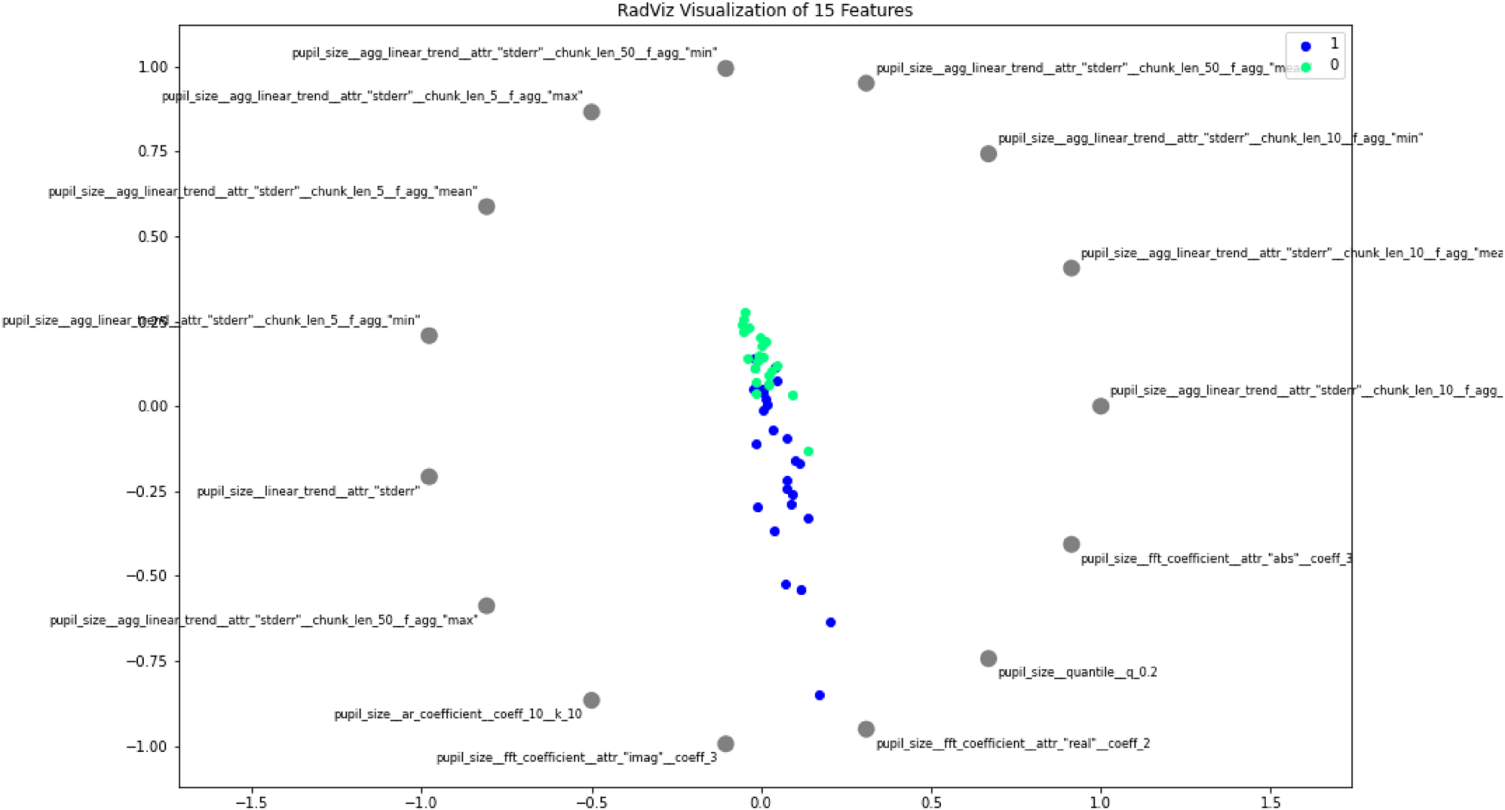
RadViz visualization of filtered features.

**Figure 5:**
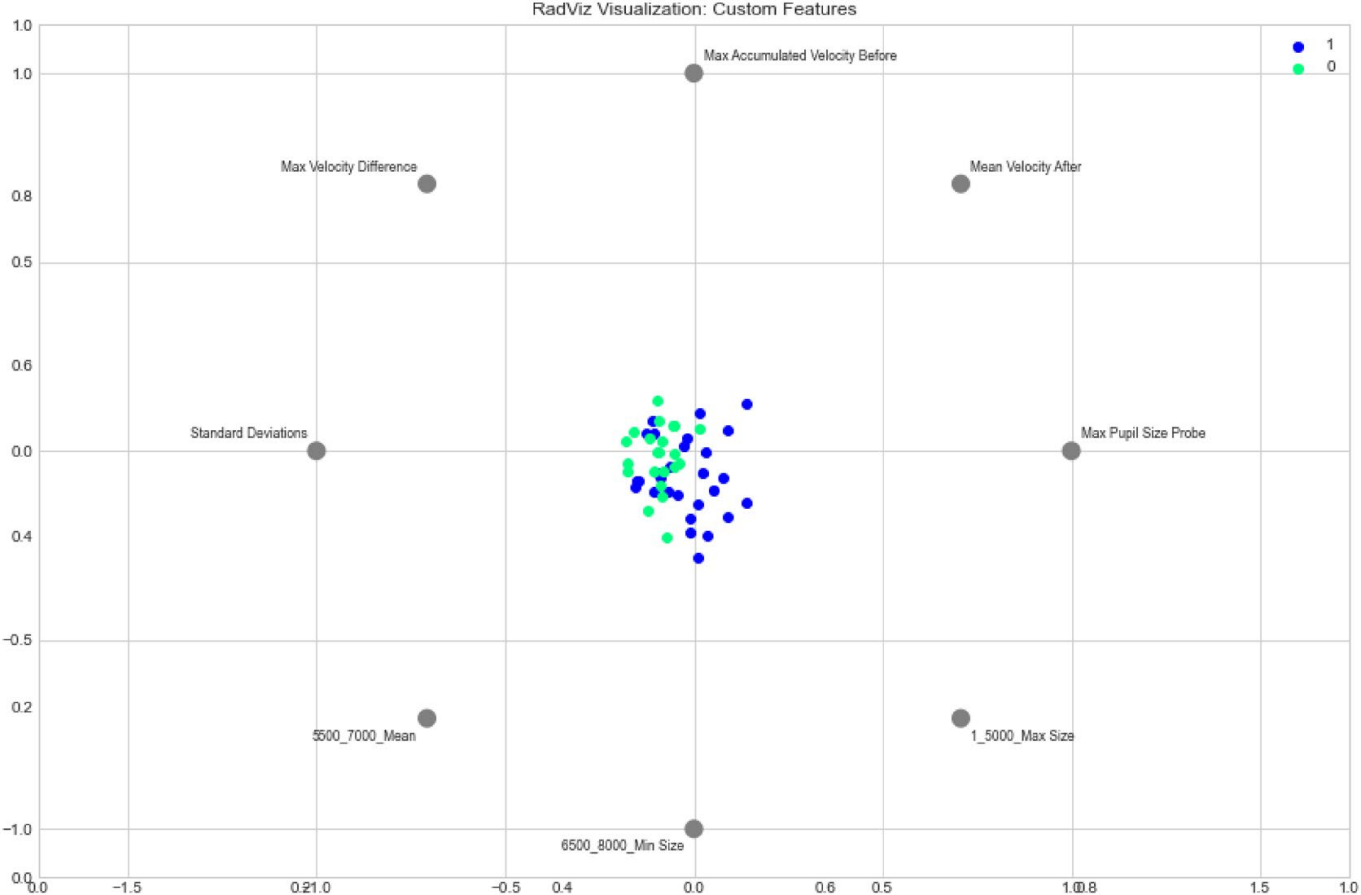
RadViz visualization of statistically significant custom features.

In order to garner intuition on discriminative pupillometric characteristics for ADHD Positive and Negative subjects, a number of features were ranked using a nonparametric Mann–Whitney U Test^29^. The top 15 out of the 783 ranking features were selected and visualized in order to examine their strengths in differentiating between ADHD positive and negative subjects. We present class separability visualizations using the RadViz multivariate visualization algorithm, which projects a feature space onto a 2D plane in order to observe class separability among various features, grouped by ADHD diagnosis^30^. Features are placed uniformly around the circumference of a circle, with data points placed in the interior of the circle—the position of a given data point gravitates proportionally in the direction towards the corresponding features based on its values, as shown in Figs. 4 and 5; data points that gravitate or cluster towards a certain feature can provide insights into discriminative pupillometric characteristics that are unique to each class. RadViz, thus, is primarily used by data scientists to examine class separability among features between classes. 15 optimal features, as well as statistically significant custom features, were separately visualized. The features visualized through RadViz were not features selected for use in machine learning model evaluation, as this would severely overfit the training data and leak information from the test sets in the Nested CV folds if features were to be filtered from the entire dataset—rather, features were selected separately on the train data during each fold.

### Statistical Tests

For each engineered feature, a nonparametric Mann–Whitney U test was used to to quantitatively assess how effective the generated features were in differentiating between ADHD positive and negative groups, where ADHD positive represents only *Off-ADHD* ground truth labels. We present a list of p-values and feature names in a supplemental file.

### Implementation Details

All the machine learning algorithms were implemented in Scikit-learn 0.22.2^31^. Feature extraction was implemented using the tsfresh 0.16.0 library^32^.

## Results

### Feature Visualization

#### Line Plots

Figures 1, 2, and 3 illustrate time-series line plots for the mean pupil size diameter, as well as pupil size dilation velocity and acceleration, grouped by diagnosis of ADHD. As can be visually observed, control subjects exhibit greater dilation velocity and acceleration, and overall variation in pupil-size. Moreover, the maximum pupil size after the presentation of a probe is noticeably larger for control subjects. The minimum pupil size from the 7s mark is noticeably smaller for control subjects.

#### Box and Violin Plots

**Figure 6:**
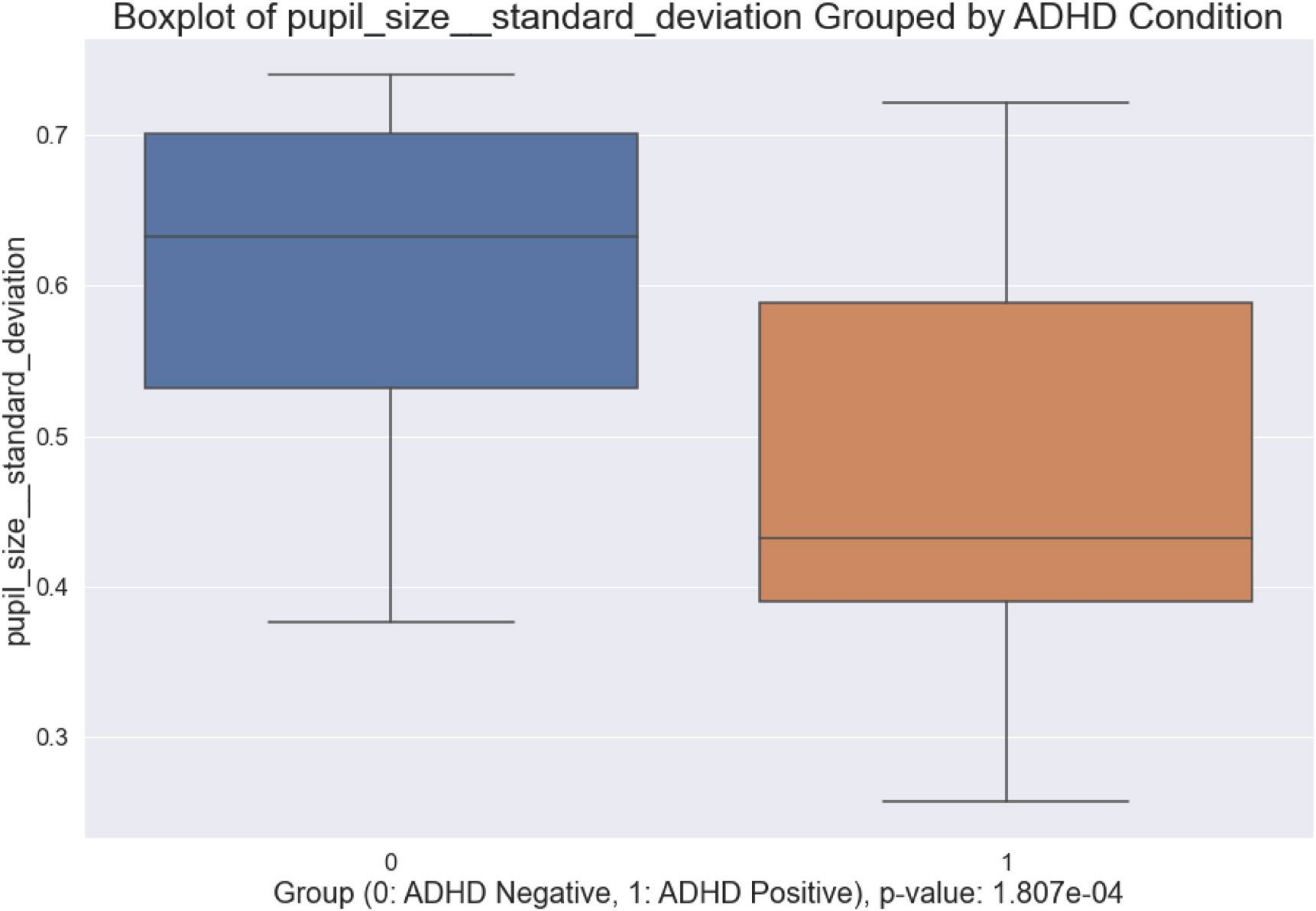
Box plot of pupil-size standard deviation.

**Figure 7:**
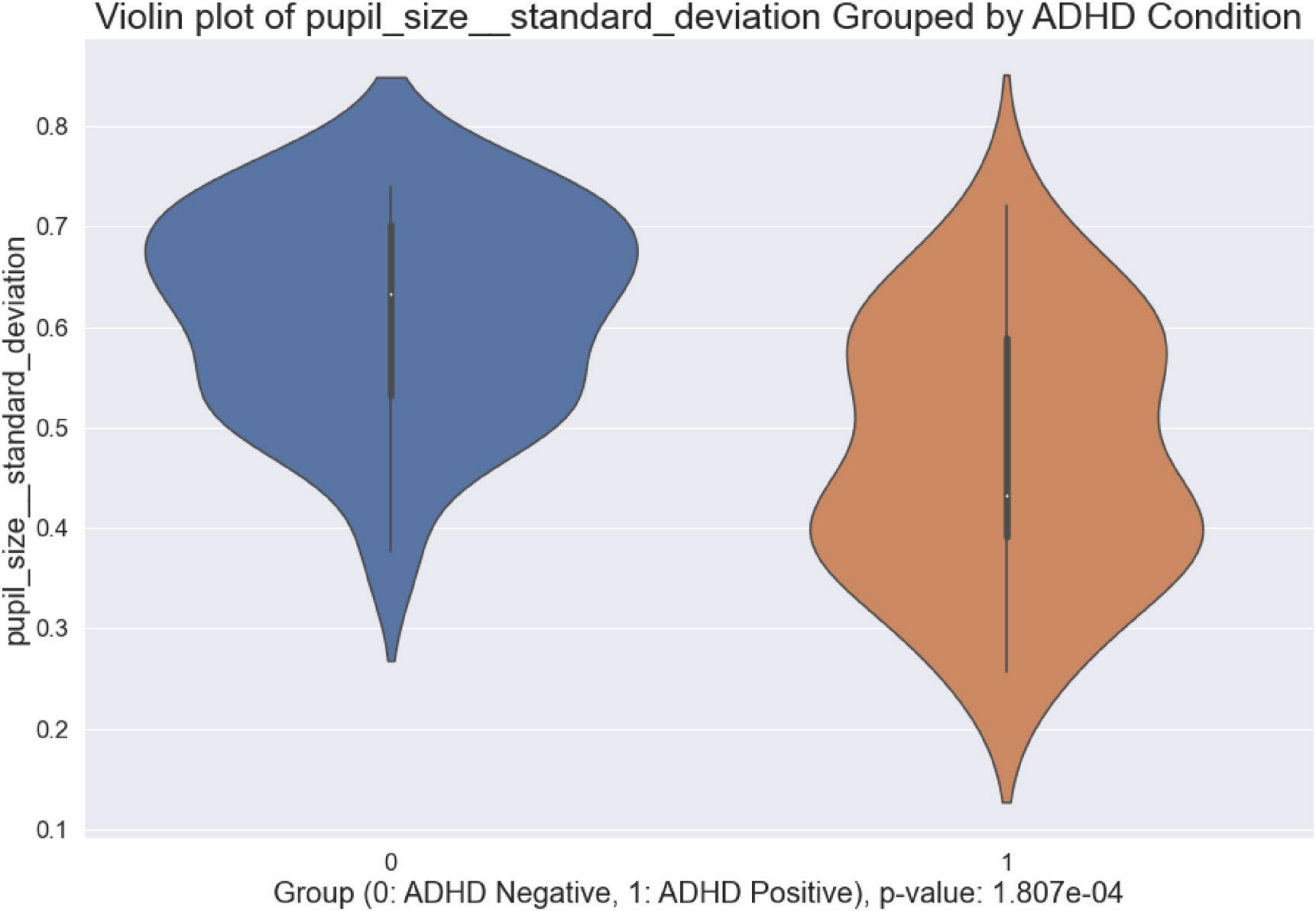
Violin plot of pupil-size standard deviation.

Figures 6 and 7 illustrate box and violin plots for pupil-size standard deviation, one statistically significant engineered feature ($p < 0.001$) that was validated statistically after visual observations. The exact p-value is shown in the figure. The distribution of standard deviation in pupil-size for control subjects overlaps with that of ADHD positive subjects, but the middle 50% of data and median values differ substantially. The distribution of values per-class illustrates that variation in pupillometrics strongly differs between ADHD Positive and Negative groups, with healthy subjects exhibiting greater standard deviation and mobility.

#### RadViz Class Separability

Figure 4 shows strong class separability between ADHD Positive and Negative subjects, with a slight overlap between the two classes using the 15 ranked features. Visually, the ADHD Positive and Negative classes are attracted to opposite spectrums of the RadViz plot, with very little overlap between the classes. The ADHD Positive data points orient themselves more toward the bottom, while ADHD Negative data points are predominantly drawn toward the top, indicating that these filtered features do enable strong separability. More importantly, the strong class separability shows that applying efficient and accurate machine learning using features derived from the encompassing set is possible using binary classification algorithms.

In Fig. 5, for the custom features, the ADHD Positive Class predominantly forms a cluster of its own—however, the overlap between the two classes is more apparent, and the ADHD Negative class does not separate as much from the Positive Class as in Fig. 4.

### Significant Features

Among the custom engineered features, the mean velocity after the presentation of a stimulus, max accumulated velocity before the stimulus, and the difference in max velocities before and after probe presentation had p-values of 4.2e−02, 4.2e−02, and 4.9e−02, respectively. The additional significant features are listed in the RadViz visualization in Fig. 5. Median aggregated values for each feature, grouped by ADHD diagnosis, are shown in Table 2. The mean velocity following probe presentation is larger for healthy subjects, as well as the overall difference in maximum velocity attained. ADHD Positive subjects on average, however, show a larger maximum accumulated velocity before the presentation of a probe.

**Table 2:** Pupil-size statistics for significant custom features.

Group	Mean Velocity After	Max Accumulated Velocity Before	Max Velocity Difference
0	0.0162	0.188	0.0397
1	0.0128	0.208	0.0351

For the rest of the engineered features, we present names of features and p-values in a supplemental file, and report a select few of the filtered features that were statistically significant, which are showed in Table 3. Standard deviation, real values of Fourier coefficients, the sum of squares of a series (absolute energy), variation in consecutive pupil-size changes during quantiles 0.4–0.8, approximate entropy of the time series with comparative lengths of 2 timestamps, and the number of peaks relative to 10 neighboring timestamps, yielded p-values of 1.8e−04, 1.8e−04, 7.2e−05, 1.8e−04, 8.5e−04, 9.1e−04, and 2.6e−04, respectively. We aggregated these features per-group based on median values for each statistic, presented in Table 3. For the Fourier coefficients, denoted by FFT (Fast Fourier Transform), the first value in parentheses corresponds to the type of value extracted, followed by a number which corresponds to the *i*th coefficient. The quantile change is recorded from quantile 0.4–0.8, where the variance of absolute changes in pupil-size at consecutive intervals was calculated. The number of peaks is calculated by counting the number of occurrences in which a given pupil-size is greater than its 10 neighboring values on the left and right side.

**Table 3:** Pupil-size statistics for significant features.

Group	$\sigma$	Absolute Energy	Standard Error of Linear Trend	FFT Coeff (Real, 2)	Quantile Change (0.4–0.8)	Approximate Entropy	Peak Number (n = 10)
0	0.633	3110.85	0.000937	− 1579.08	0.000002	0.001782	41
1	0.432	1927.64	0.000706	− 1058.30	0.000004	0.002417	71

As shown in Table 3, ADHD Positive subjects exhibit lower standard deviation, absolute energy, and standard error for regression coefficients. They exhibit greater approximate entropy, quantile changes, as well as overall peak numbers throughout the time-series.

### Classifier Results

#### Binary Classification Metrics

State-of-the-art machine learning algorithms were evaluated based on three key medical diagnostic classification metrics: sensitivity, specificity, and AUROC. An evaluation of the models is shown in Table 4. The Naive Bayes classifier achieved the highest AUROC and specificity, but exhibited low sensitivity metrics. The Logistic Regression classifier yielded the highest sensitivity, coupled with a moderate balance with specificity and a strong AUROC. Furthermore, the support vector machine achieved an excellent AUROC, with a strong balance in sensitivity and specificity.

**Table 4:** Binary classifier nested ten-fold CV metrics.

Model	Sensitivity (TPR)	Specificity (TNR)	AUROC	Accuracy
Logistic Regression	0.794 (± 0.020)	0.715 (± 0.028)	0.867 (± 0.015)	0.759 (± 0.015)
Support Vector Machine	0.773 (± 0.022)	0.753 (± 0.027)	0.856 (± 0.016)	0.762 (± 0.015)
Decision Tree	0.787 (± 0.022)	0.641 (± 0.029)	0.758 (± 0.018)	0.725 (± 0.016)
Naive Bayes	0.656 (± 0.025)	0.863 (± 0.020)	0.871 (± 0.015)	0.749 (± 0.015)
KNN Neighbors	0.830 (± 0.017)	0.752 (± 0.026)	0.830 (± 0.017)	0.724 (± 0.016)
Random Forest Classifier	0.786 (± 0.021)	0.701 (± 0.027)	0.832 (± 0.017)	0.750 (± 0.016)

#### ROC Analysis

**Figure 8:**
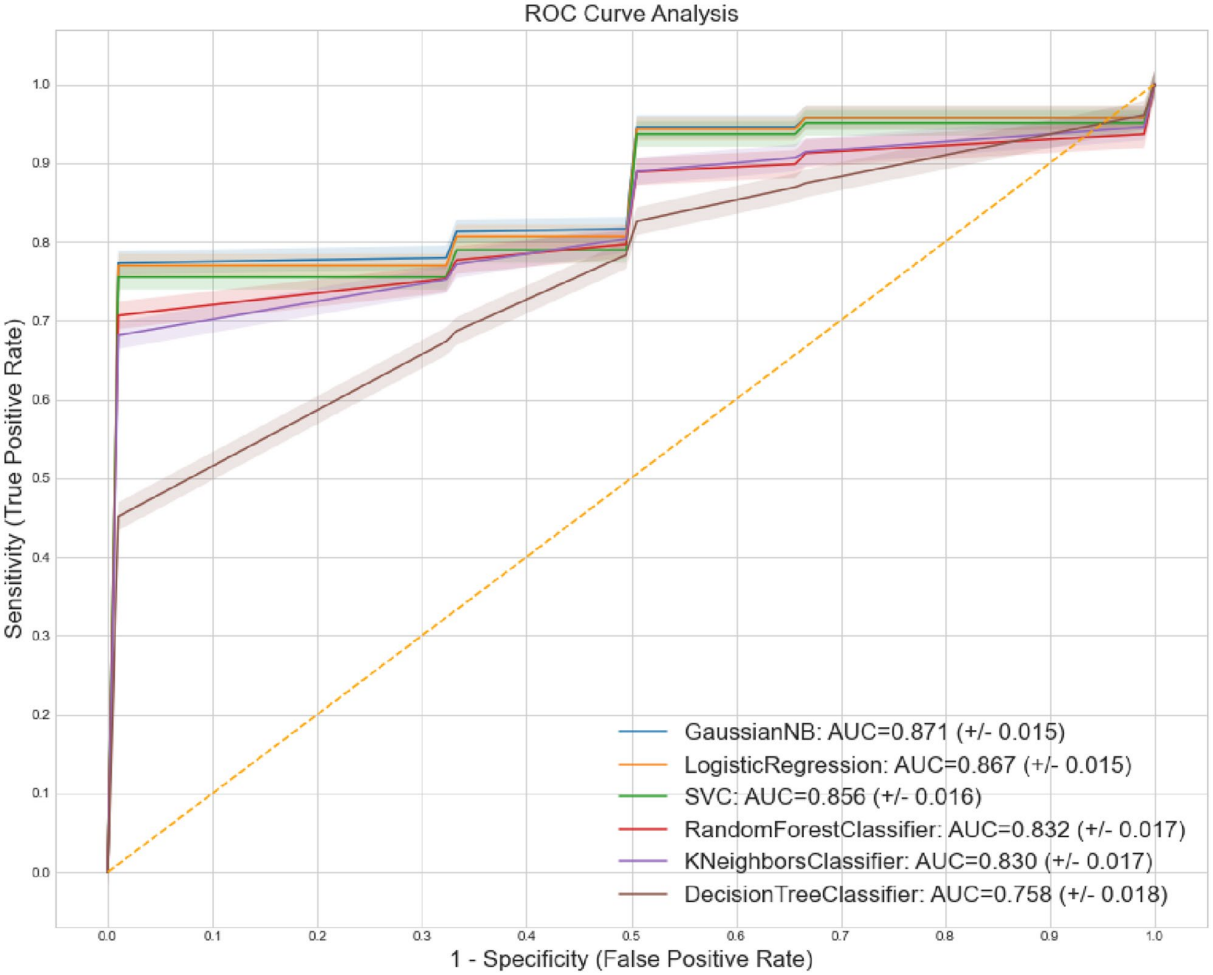
Nested CV ROC analysis of binary classifiers.

Figure 8 illustrates the ROC curves for all binary machine learning classifiers tested using ten-fold nested CV.

## Discussion

### Machine Learning Robustness

This study utilized a small dataset of 50 instances for machine learning model evaluation—for this reason, ten-fold nested CV was applied to the data to best assess how well each of the models could generalize to unseen data. Bayesian methods and simple classifiers like Logistic Regression tend to perform well on small datasets, which is reflected in our results. The support vector machine classifier, howeover, seems most promising given the strong balance in sensitivity and specificity, as well as an excellent AUROC of 0.856 (± 0.16). We note that the small dataset size significantly skews the sensitivity and specificity metrics, but the overall excellent AUROC metrics indicate that the classifiers are robust in detecting ADHD—as such, with additional clinical data, sensitivity and specificity metrics can be substantially augmented, enabling high-accuracy and efficient detection of ADHD. In small datasets, AUROC can assist in providing a more comprehensive assessment of binary classification ability by measuring accuracy at varying thresholds and taking into account probabilistic scores rather than class label predictions. The excellent AUROC values of the classifiers presented improve upon the reported misdiagnosis rate to be approximately 80% in 2010—approximately 20 percent of 4.5 million children using stimulant prescription medication were found to be misdiagnosed from a research study^7^. Additional data, however, is needed to adequately assess sensitivity, specificity, and accuracy using a holdout test dataset. Overall, howeover, the strong AUROC values indicate the potential to achieve very strong metrics for the detection of ADHD with additional data, and provide a proof-of-concept that highlights the potential to use pupillometrics to effectively differentiate between ADHD Positive and Negative subjects.

Moreover, the statistically significant features provide a strong foundation for the binary classifiers and a new lens to view relationships in pupil-size dynamics and the presence of ADHD, reflected in our statistical validation and class separability visualizations. The features overall exhibited strong class separability between ADHD Positive and Negative groups, which is reflected in the AUROC values of the classifiers.

We note that one drawback of our study lies in the small sample size, which can reasonably be only generalized to the encompassing city in Chile from which the data was collected. Additional data would help in creating a sample representative of the true population.

### New Behavioral Understandings

Insights into the pupil-size dynamics of ADHD positive and negative subjects were uncovered. In particular, variation, specifically standard deviation, in pupil-size dilation velocity, previously not studied within the scope of ADHD, were found to be strong discriminators between ADHD positive and negative subjects. Control subjects visually exhibited greater pupil dilation velocity and acceleration compared to ADHD positive subjects, indicative of heightened neural activity and decision making. ADHD positive subjects exhibit lower dilation speeds and pupil sizes most likely because of uncertainty and inattentiveness—Van de Brink et al. highlighted that smaller relative pupil size linearly correlates with greater lapses in attention, while greater pupil sizes correlate with increased attentional effort^33^. The statistical significance of the engineered features in this study further illustrate its validity and strength in differentiating between ADHD positive and negative groups. Our results also show that local maxima and minima in pupil-size are also strong differentiators between positive and negative subjects. Moreover, standard deviation in pupil-size across a time-series was found to be a strong discriminator between the two groups, with control subjects exhibiting greater variation in pupil-size, indicating that ADHD positive subjects potentially suffer from lack of overall mobility in pupillary responses, which could further inform the use of pupillometrics as a biomarker to characterize ADHD.

Additionally, absolute energy values, consecutive quantile changes, approximate entropy metrics, and peak numbers provide informative metrics for potentially differentiating between ADHD Positive and Negative subjects. Healthy subjects exhibit greater overall energy, or sum of squares, across a time-series, which indicates that they generally exhibit greater pupil-sizes across a trial than ADHD positive subjects, which concurs with the notion that larger pupil-sizes represent increased attentional effort^33^. ADHD Positive subjects, however, exhibit greater variance in consecutive changes in pupil-size, measured in absolute value, from quantile 0.4 to 0.8, which includes the presentation of a stimulus. This suggests that their pupillometric patterns are more erratic when measured at consecutive timestamps, especially during the presentation of a probe, as opposed to more fluid changes in pupil-size exhibited by control subjects at consecutive timestamps. In accordance with this, approximate entropy, which measures regularity and unpredictability across a time-series^34^, is higher for ADHD positive subjects, further suggesting that their pupillary movements are more erratic than healthy subjects. The larger number of peak sizes also concurs this observation among ADHD Positive subjects.

### Applications and Potential

Our machine learning based framework offers a novel and reliable technical approach to diagnose ADHD that is time-efficient and reliant on an objective biomarker, rather than inaccurate, subjective clinical evaluations. Evaluations currently last multiple hours and rely on loose qualitative observations^6^. Our findings can streamline clinical diagnosis and serve as a novel lens to view associations between pupillometrics and the presence of ADHD using machine learning. Moreover, they can enable medical professionals to make more informed decisions for diagnosing ADHD accurately. We provide a proof-of-concept, showing that pupillometrics can be used to effectively differentiate between ADHD Positive and Negative groups using machine learning. Future work to increase model performance and robustness revolve around attaining additional clinical and data and potentially integrating eye gaze directions with pupillometrics to provide a comprehensive and more accurate risk score of having ADHD. In conjunction with this, deep learning based models could enable more scalable and powerful analysis of this oculometric data for ADHD detection in the future with additional data. Moreover, potentially employing a multi-classification model that takes into account *On-ADHD*, *Off-ADHD*, and *Ctrl* subjects could enable a more robust framework.

## Supplementary Information

Supplementary Information.
