# Supplementary material for: A Robust Machine Learning Based Framework for the Automated Detection of ADHD Using Pupillometric Biomarkers and Time Series Analysis
Source: Sci Rep. 2021 Aug 12;11:16370. doi: 10.1038/s41598-021-95673-5 (PMC8361128; doi:10.1038/s41598-021-95673-5)
Supplement: Supplementary file 1 — Supplementary Information. [file 41598_2021_95673_MOESM1_ESM.docx]

**A Robust Machine Learning Based Framework for the Automated Detection of ADHD Using Pupillometric Biomarkers and Time Series Analysis**

William Das and Shubh Khanna

**Side-by-side Plot of Reduced Time Series Data**

**
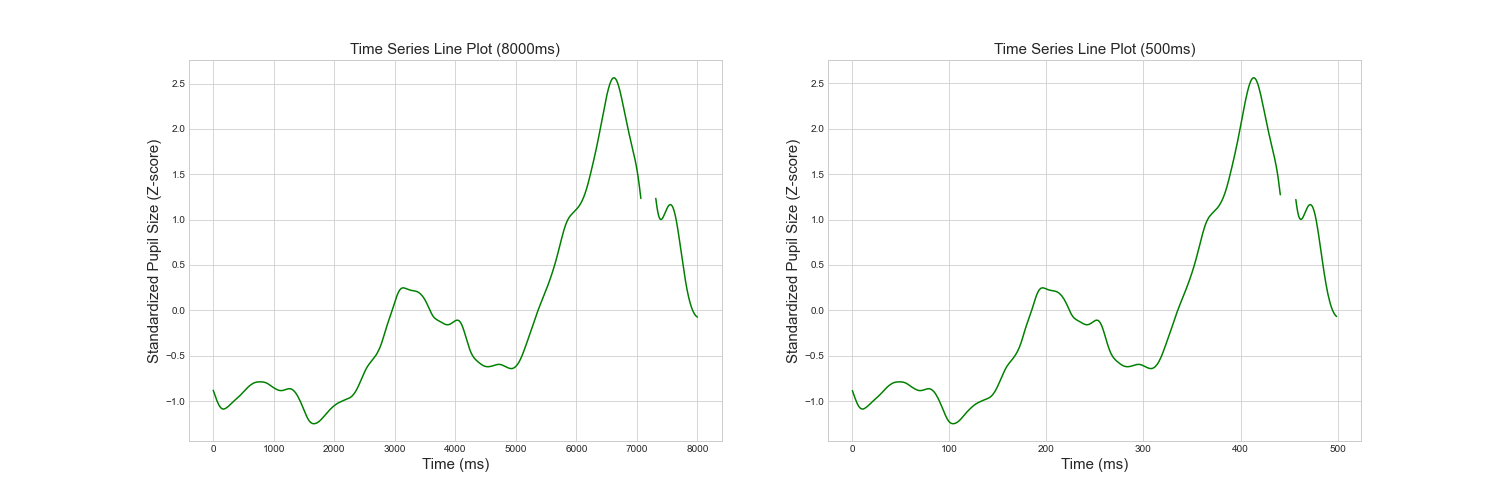
**

**Side-by-side plot of reduced time-series data for one trial.**

**Table of Engineered Features and p-values:**

**Feature, p-value**

"pupil_size__agg_linear_trend__attr_""stderr""__chunk_len_10__f_agg_""max""",2.514885592816328e-06

"pupil_size__agg_linear_trend__attr_""stderr""__chunk_len_10__f_agg_""mean""",2.514885592816328e-06

"pupil_size__agg_linear_trend__attr_""stderr""__chunk_len_10__f_agg_""min""",2.514885592816328e-06

"pupil_size__agg_linear_trend__attr_""stderr""__chunk_len_50__f_agg_""mean""",2.514885592816328e-06

"pupil_size__agg_linear_trend__attr_""stderr""__chunk_len_50__f_agg_""min""",2.514885592816328e-06

"pupil_size__agg_linear_trend__attr_""stderr""__chunk_len_5__f_agg_""max""",2.514885592816328e-06

"pupil_size__agg_linear_trend__attr_""stderr""__chunk_len_5__f_agg_""mean""",2.514885592816328e-06

"pupil_size__agg_linear_trend__attr_""stderr""__chunk_len_5__f_agg_""min""",2.514885592816328e-06

"pupil_size__linear_trend__attr_""stderr""",2.514885592816328e-06

"pupil_size__agg_linear_trend__attr_""stderr""__chunk_len_50__f_agg_""max""",3.027581447600593e-06

pupil_size__ar_coefficient__coeff_10__k_10,2.7202249026556776e-05

"pupil_size__fft_coefficient__attr_""imag""__coeff_3",3.2107147715931544e-05

"pupil_size__fft_coefficient__attr_""real""__coeff_2",7.196273905845381e-05

pupil_size__quantile__q_0.2,9.837752598958581e-05

"pupil_size__fft_coefficient__attr_""abs""__coeff_3",0.00010627953136443615

pupil_size__quantile__q_0.1,0.00010627953136443615

pupil_size__abs_energy,0.00018068755285729278

pupil_size__standard_deviation,0.00018068755285729278

pupil_size__variance,0.00018068755285729278

pupil_size__cid_ce__normalize_True,0.00024276236478635907

pupil_size__number_peaks__n_10,0.00026862375071424295

"pupil_size__fft_aggregated__aggtype_""skew""",0.00037399919896092594

pupil_size__number_peaks__n_50,0.0003806828774369883

"pupil_size__fft_aggregated__aggtype_""centroid""",0.0004014264857053385

"pupil_size__fft_aggregated__aggtype_""kurtosis""",0.0004014264857053385

pupil_size__maximum,0.0004014264857053385

"pupil_size__fft_coefficient__attr_""abs""__coeff_28",0.000430710574407695

"pupil_size__fft_aggregated__aggtype_""variance""",0.0004619652285732096

"pupil_size__fft_coefficient__attr_""abs""__coeff_2",0.0004953103113043276

pupil_size__number_peaks__n_5,0.0005301341519460418

"pupil_size__fft_coefficient__attr_""real""__coeff_4",0.0005308720525936784

Max Pupil Size Probe,0.0005687833244458051

5500_7000_Mean,0.0006522208632629725

pupil_size__ar_coefficient__coeff_8__k_10,0.0006522208632629725

pupil_size__minimum,0.0007468296890426835

"pupil_size__change_quantiles__f_agg_""var""__isabs_True__qh_0.8__ql_0.2",0.0007987344023720484

"pupil_size__change_quantiles__f_agg_""var""__isabs_True__qh_0.8__ql_0.4",0.0008539417442727181

pupil_size__approximate_entropy__m_2__r_0.7,0.0009126394322708155

pupil_size__number_peaks__n_3,0.0009426441035470637

"pupil_size__fft_coefficient__attr_""abs""__coeff_4",0.001041302648045184

pupil_size__approximate_entropy__m_2__r_0.5,0.0011116905748856589

pupil_size__range_count__max_1__min_-1,0.0011428724928345146

"pupil_size__fft_coefficient__attr_""imag""__coeff_18",0.0012657107632456101

pupil_size__spkt_welch_density__coeff_8,0.0012657107632456101

"pupil_size__change_quantiles__f_agg_""var""__isabs_True__qh_1.0__ql_0.2",0.001439023500658101

"pupil_size__agg_linear_trend__attr_""stderr""__chunk_len_50__f_agg_""var""",0.001533569354006235

pupil_size__approximate_entropy__m_2__r_0.1,0.001533569354006235

pupil_size__spkt_welch_density__coeff_5,0.001633747663944412

Standard Deviations,0.0018521944924416156

"pupil_size__change_quantiles__f_agg_""var""__isabs_True__qh_0.6__ql_0.4",0.0018521944924416156

pupil_size__quantile__q_0.9,0.001971091807859896

pupil_size__approximate_entropy__m_2__r_0.3,0.002519147135268246

pupil_size__number_cwt_peaks__n_1,0.002840736690345294

"pupil_size__change_quantiles__f_agg_""var""__isabs_True__qh_0.6__ql_0.2",0.0028418936650329634

pupil_size__energy_ratio_by_chunks__num_segments_10__segment_focus_7,0.0028418936650329634

"pupil_size__fft_coefficient__attr_""abs""__coeff_73",0.003201478921566381

"pupil_size__change_quantiles__f_agg_""var""__isabs_True__qh_1.0__ql_0.4",0.00339619841569297

pupil_size__quantile__q_0.3,0.003817864158362578

"pupil_size__fft_coefficient__attr_""imag""__coeff_11",0.004045812274307253

"pupil_size__change_quantiles__f_agg_""var""__isabs_False__qh_0.8__ql_0.2",0.004804511076227709

"pupil_size__fft_coefficient__attr_""abs""__coeff_29",0.004804511076227709

"pupil_size__fft_coefficient__attr_""abs""__coeff_81",0.004804511076227709

"pupil_size__fft_coefficient__attr_""abs""__coeff_90",0.004804511076227709

"pupil_size__fft_coefficient__attr_""abs""__coeff_97",0.005084247541598376

"pupil_size__augmented_dickey_fuller__attr_""pvalue""__autolag_""AIC""",0.005378393337454459

"pupil_size__augmented_dickey_fuller__attr_""teststat""__autolag_""AIC""",0.005378393337454459

"pupil_size__change_quantiles__f_agg_""var""__isabs_False__qh_1.0__ql_0.2",0.005378393337454459

"pupil_size__change_quantiles__f_agg_""var""__isabs_True__qh_0.8__ql_0.6",0.005378393337454459

"pupil_size__fft_coefficient__attr_""real""__coeff_95",0.005378393337454459

pupil_size__number_cwt_peaks__n_5,0.005523347017428473

pupil_size__approximate_entropy__m_2__r_0.9,0.005687572577533766

"pupil_size__fft_coefficient__attr_""abs""__coeff_25",0.005687572577533766

"pupil_size__change_quantiles__f_agg_""var""__isabs_False__qh_0.8__ql_0.4",0.006012429497751323

"pupil_size__fft_coefficient__attr_""abs""__coeff_0",0.006012429497751323

pupil_size__c3__lag_1,0.0063536287238014424

pupil_size__c3__lag_2,0.0063536287238014424

pupil_size__c3__lag_3,0.0063536287238014424

"pupil_size__fft_coefficient__attr_""imag""__coeff_55",0.006711855520401172

pupil_size__spkt_welch_density__coeff_2,0.006711855520401172

pupil_size__number_peaks__n_1,0.006894162676293925

pupil_size__number_crossing_m__m_-1,0.007176127247658935

"pupil_size__change_quantiles__f_agg_""var""__isabs_True__qh_1.0__ql_0.6",0.007895868234926336

"pupil_size__fft_coefficient__attr_""abs""__coeff_26",0.007895868234926336

"pupil_size__fft_coefficient__attr_""angle""__coeff_73",0.007895868234926336

"pupil_size__change_quantiles__f_agg_""var""__isabs_True__qh_0.8__ql_0.0",0.008329478317074585

"pupil_size__fft_coefficient__attr_""abs""__coeff_11",0.008329478317074585

"pupil_size__fft_coefficient__attr_""abs""__coeff_72",0.008329478317074585

pupil_size__sample_entropy,0.008329478317074585

"pupil_size__change_quantiles__f_agg_""var""__isabs_True__qh_1.0__ql_0.0",0.008783859136866521

"pupil_size__fft_coefficient__attr_""real""__coeff_51",0.008783859136866521

"pupil_size__fft_coefficient__attr_""abs""__coeff_63",0.00925982381803236

pupil_size__max_langevin_fixed_point__m_3__r_30,0.01082567875491321

"pupil_size__fft_coefficient__attr_""real""__coeff_20",0.011396545634499135

"pupil_size__fft_coefficient__attr_""abs""__coeff_18",0.012617151726555743

"pupil_size__fft_coefficient__attr_""angle""__coeff_84",0.012617151726555743

"pupil_size__fft_coefficient__attr_""real""__coeff_1",0.012617151726555743

"pupil_size__fft_coefficient__attr_""real""__coeff_52",0.012617151726555743

pupil_size__number_crossing_m__m_1,0.012939879940710957

"pupil_size__change_quantiles__f_agg_""var""__isabs_False__qh_0.6__ql_0.4",0.013268799880677813

"pupil_size__fft_coefficient__attr_""abs""__coeff_68",0.013268799880677813

pupil_size__ar_coefficient__coeff_4__k_10,0.013949319804955165

pupil_size__ar_coefficient__coeff_7__k_10,0.013949319804955165

"pupil_size__change_quantiles__f_agg_""var""__isabs_False__qh_1.0__ql_0.0",0.013949319804955165

pupil_size__cid_ce__normalize_False,0.013949319804955165

"pupil_size__fft_coefficient__attr_""abs""__coeff_13",0.013949319804955165

"pupil_size__fft_coefficient__attr_""abs""__coeff_64",0.014659719290087504

1_5000_Max Size,0.015401027053631673

"pupil_size__fft_coefficient__attr_""abs""__coeff_30",0.015401027053631673

"pupil_size__fft_coefficient__attr_""angle""__coeff_15",0.01698058513857589

"pupil_size__fft_coefficient__attr_""imag""__coeff_4",0.01698058513857589

"pupil_size__change_quantiles__f_agg_""var""__isabs_False__qh_0.8__ql_0.0",0.01782099469845952

"pupil_size__cwt_coefficients__coeff_6__w_2__widths_(2, 5, 10, 20)",0.01782099469845952

"pupil_size__fft_coefficient__attr_""abs""__coeff_1",0.01782099469845952

"pupil_size__fft_coefficient__attr_""abs""__coeff_20",0.01782099469845952

"pupil_size__fft_coefficient__attr_""abs""__coeff_35",0.01782099469845952

"pupil_size__fft_coefficient__attr_""imag""__coeff_19",0.01782099469845952

"pupil_size__change_quantiles__f_agg_""var""__isabs_False__qh_0.6__ql_0.2",0.01869663020560687

"pupil_size__fft_coefficient__attr_""abs""__coeff_71",0.01869663020560687

"pupil_size__fft_coefficient__attr_""angle""__coeff_72",0.01869663020560687

"pupil_size__fft_coefficient__attr_""imag""__coeff_20",0.01869663020560687

"pupil_size__fft_coefficient__attr_""real""__coeff_70",0.01869663020560687

"pupil_size__change_quantiles__f_agg_""var""__isabs_False__qh_1.0__ql_0.4",0.01960861975418571

pupil_size__energy_ratio_by_chunks__num_segments_10__segment_focus_5,0.01960861975418571

"pupil_size__fft_coefficient__attr_""angle""__coeff_1",0.01960861975418571

pupil_size__first_location_of_maximum,0.02007621137991582

pupil_size__last_location_of_maximum,0.02007621137991582

"pupil_size__cwt_coefficients__coeff_1__w_20__widths_(2, 5, 10, 20)",0.020558109931233946

"pupil_size__fft_coefficient__attr_""abs""__coeff_9",0.020558109931233946

"pupil_size__agg_linear_trend__attr_""slope""__chunk_len_50__f_agg_""var""",0.02154626524017594

"pupil_size__cwt_coefficients__coeff_2__w_20__widths_(2, 5, 10, 20)",0.02154626524017594

"pupil_size__fft_coefficient__attr_""abs""__coeff_92",0.02154626524017594

"pupil_size__fft_coefficient__attr_""angle""__coeff_23",0.02154626524017594

"pupil_size__cwt_coefficients__coeff_14__w_5__widths_(2, 5, 10, 20)",0.022574267472712944

"pupil_size__cwt_coefficients__coeff_4__w_20__widths_(2, 5, 10, 20)",0.022574267472712944

"pupil_size__fft_coefficient__attr_""angle""__coeff_53",0.022574267472712944

"pupil_size__cwt_coefficients__coeff_11__w_10__widths_(2, 5, 10, 20)",0.023643315028198723

"pupil_size__cwt_coefficients__coeff_12__w_10__widths_(2, 5, 10, 20)",0.023643315028198723

"pupil_size__cwt_coefficients__coeff_13__w_10__widths_(2, 5, 10, 20)",0.023643315028198723

"pupil_size__cwt_coefficients__coeff_5__w_2__widths_(2, 5, 10, 20)",0.023643315028198723

"pupil_size__cwt_coefficients__coeff_6__w_20__widths_(2, 5, 10, 20)",0.023643315028198723

"pupil_size__cwt_coefficients__coeff_9__w_20__widths_(2, 5, 10, 20)",0.023643315028198723

"pupil_size__fft_coefficient__attr_""abs""__coeff_96",0.023643315028198723

"pupil_size__fft_coefficient__attr_""imag""__coeff_31",0.023643315028198723

"pupil_size__cwt_coefficients__coeff_10__w_10__widths_(2, 5, 10, 20)",0.024754622179698597

"pupil_size__cwt_coefficients__coeff_10__w_20__widths_(2, 5, 10, 20)",0.024754622179698597

"pupil_size__cwt_coefficients__coeff_2__w_10__widths_(2, 5, 10, 20)",0.024754622179698597

"pupil_size__cwt_coefficients__coeff_3__w_20__widths_(2, 5, 10, 20)",0.024754622179698597

"pupil_size__cwt_coefficients__coeff_4__w_2__widths_(2, 5, 10, 20)",0.024754622179698597

"pupil_size__cwt_coefficients__coeff_8__w_20__widths_(2, 5, 10, 20)",0.024754622179698597

"pupil_size__fft_coefficient__attr_""angle""__coeff_42",0.024754622179698597

"pupil_size__fft_coefficient__attr_""angle""__coeff_94",0.024754622179698597

"pupil_size__cwt_coefficients__coeff_11__w_5__widths_(2, 5, 10, 20)",0.0259094182860216

"pupil_size__cwt_coefficients__coeff_12__w_5__widths_(2, 5, 10, 20)",0.0259094182860216

"pupil_size__cwt_coefficients__coeff_1__w_10__widths_(2, 5, 10, 20)",0.0259094182860216

"pupil_size__cwt_coefficients__coeff_7__w_20__widths_(2, 5, 10, 20)",0.0259094182860216

"pupil_size__cwt_coefficients__coeff_9__w_10__widths_(2, 5, 10, 20)",0.0259094182860216

"pupil_size__fft_coefficient__attr_""abs""__coeff_34",0.0259094182860216

"pupil_size__change_quantiles__f_agg_""var""__isabs_False__qh_0.8__ql_0.6",0.02710894694911387

"pupil_size__cwt_coefficients__coeff_11__w_20__widths_(2, 5, 10, 20)",0.02710894694911387

"pupil_size__cwt_coefficients__coeff_14__w_10__widths_(2, 5, 10, 20)",0.02710894694911387

"pupil_size__cwt_coefficients__coeff_3__w_10__widths_(2, 5, 10, 20)",0.02710894694911387

"pupil_size__cwt_coefficients__coeff_3__w_2__widths_(2, 5, 10, 20)",0.02710894694911387

"pupil_size__cwt_coefficients__coeff_4__w_10__widths_(2, 5, 10, 20)",0.02710894694911387

"pupil_size__cwt_coefficients__coeff_5__w_20__widths_(2, 5, 10, 20)",0.02710894694911387

"pupil_size__cwt_coefficients__coeff_7__w_5__widths_(2, 5, 10, 20)",0.02710894694911387

"pupil_size__cwt_coefficients__coeff_8__w_10__widths_(2, 5, 10, 20)",0.02710894694911387

"pupil_size__fft_coefficient__attr_""imag""__coeff_1",0.02710894694911387

"pupil_size__agg_linear_trend__attr_""rvalue""__chunk_len_5__f_agg_""var""",0.02835446511630479

"pupil_size__cwt_coefficients__coeff_12__w_20__widths_(2, 5, 10, 20)",0.02835446511630479

"pupil_size__cwt_coefficients__coeff_6__w_10__widths_(2, 5, 10, 20)",0.02835446511630479

"pupil_size__cwt_coefficients__coeff_7__w_10__widths_(2, 5, 10, 20)",0.02835446511630479

"pupil_size__cwt_coefficients__coeff_8__w_5__widths_(2, 5, 10, 20)",0.02835446511630479

"pupil_size__cwt_coefficients__coeff_9__w_5__widths_(2, 5, 10, 20)",0.02835446511630479

"pupil_size__change_quantiles__f_agg_""var""__isabs_True__qh_0.4__ql_0.2",0.029647242127005664

"pupil_size__cwt_coefficients__coeff_13__w_5__widths_(2, 5, 10, 20)",0.029647242127005664

"pupil_size__cwt_coefficients__coeff_2__w_2__widths_(2, 5, 10, 20)",0.029647242127005664

"pupil_size__cwt_coefficients__coeff_4__w_5__widths_(2, 5, 10, 20)",0.029647242127005664

"pupil_size__cwt_coefficients__coeff_5__w_10__widths_(2, 5, 10, 20)",0.029647242127005664

"pupil_size__cwt_coefficients__coeff_5__w_5__widths_(2, 5, 10, 20)",0.029647242127005664

"pupil_size__cwt_coefficients__coeff_6__w_5__widths_(2, 5, 10, 20)",0.029647242127005664

"pupil_size__fft_coefficient__attr_""real""__coeff_21",0.029647242127005664

"pupil_size__fft_coefficient__attr_""real""__coeff_23",0.029647242127005664

"pupil_size__cwt_coefficients__coeff_13__w_20__widths_(2, 5, 10, 20)",0.030988558703576204

"pupil_size__cwt_coefficients__coeff_3__w_5__widths_(2, 5, 10, 20)",0.030988558703576204

"pupil_size__fft_coefficient__attr_""abs""__coeff_41",0.030988558703576204

"pupil_size__cwt_coefficients__coeff_10__w_5__widths_(2, 5, 10, 20)",0.032379705886191795

"pupil_size__cwt_coefficients__coeff_14__w_20__widths_(2, 5, 10, 20)",0.032379705886191795

"pupil_size__cwt_coefficients__coeff_1__w_2__widths_(2, 5, 10, 20)",0.032379705886191795

"pupil_size__fft_coefficient__attr_""abs""__coeff_78",0.032379705886191795

"pupil_size__fft_coefficient__attr_""abs""__coeff_95",0.032379705886191795

"pupil_size__cwt_coefficients__coeff_2__w_5__widths_(2, 5, 10, 20)",0.033821983911670635

"pupil_size__cwt_coefficients__coeff_1__w_5__widths_(2, 5, 10, 20)",0.035316701036347736

"pupil_size__fft_coefficient__attr_""abs""__coeff_93",0.035316701036347736

"pupil_size__fft_coefficient__attr_""angle""__coeff_24",0.035316701036347736

6500_8000_Min Size,0.0368651723032172

"pupil_size__fft_coefficient__attr_""abs""__coeff_27",0.0368651723032172

"pupil_size__fft_coefficient__attr_""abs""__coeff_79",0.0368651723032172

"pupil_size__fft_coefficient__attr_""abs""__coeff_42",0.038468718253702155

"pupil_size__fft_coefficient__attr_""angle""__coeff_3",0.038468718253702155

"pupil_size__fft_coefficient__attr_""real""__coeff_78",0.038468718253702155

"pupil_size__fft_coefficient__attr_""angle""__coeff_56",0.040128663584554164

Mean Velocity After,0.041846335750529394

Max Accumulated Velocity Before,0.041846335750529394

"pupil_size__change_quantiles__f_agg_""var""__isabs_True__qh_1.0__ql_0.8",0.041846335750529394

pupil_size__energy_ratio_by_chunks__num_segments_10__segment_focus_3,0.041846335750529394

"pupil_size__fft_coefficient__attr_""abs""__coeff_33",0.041846335750529394

"pupil_size__fft_coefficient__attr_""real""__coeff_79",0.041846335750529394

"pupil_size__agg_linear_trend__attr_""stderr""__chunk_len_5__f_agg_""var""",0.04362306351363877

"pupil_size__fft_coefficient__attr_""real""__coeff_72",0.04362306351363877

"pupil_size__fft_coefficient__attr_""angle""__coeff_55",0.045460175439921736

"pupil_size__fft_coefficient__attr_""angle""__coeff_86",0.045460175439921736

pupil_size__ar_coefficient__coeff_1__k_10,0.047358998344848274

"pupil_size__fft_coefficient__attr_""imag""__coeff_68",0.047358998344848274

pupil_size__index_mass_quantile__q_0.4,0.04931681060144075

Max Velocity Difference,0.04932085568861224

"pupil_size__fft_coefficient__attr_""angle""__coeff_52",0.04932085568861224

"pupil_size__fft_coefficient__attr_""angle""__coeff_9",0.04932085568861224

"pupil_size__fft_coefficient__attr_""real""__coeff_15",0.04932085568861224
